# Socio-environmental Opportunities for Organic Material Management in California’s Sustainability Transition

**DOI:** 10.1021/acs.est.3c10711

**Published:** 2024-05-16

**Authors:** Anaya L. Hall, Aleksandra I. Ponomareva, Margaret S. Torn, Matthew D. Potts

**Affiliations:** † Energy & Resources Group University of California—Berkeley 345 Giannini Hall Berkeley, California 94720, United States; ‡ Climate and Ecosystem Sciences Division Lawrence Berkeley National Laboratory Berkeley, California 94720, United States; § Department of Environmental Science, Policy, and Management University of California—Berkeley 130 Mulford Hall Berkeley, California 94720, United States; ∥ Carbon Direct, Incorporated 17 State Street New York, New York 10004, United States

**Keywords:** carbon sequestration, climate change mitigation, environmental justice, food waste, organics

## Abstract

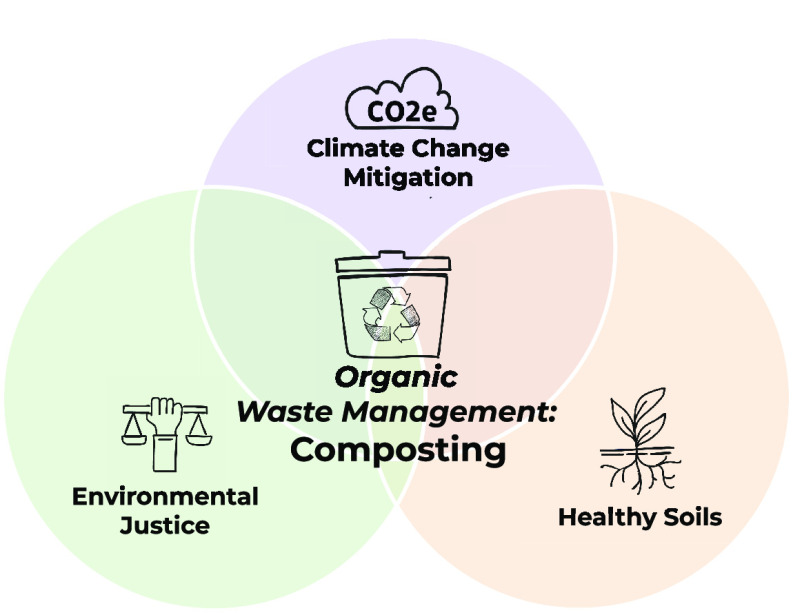

Contemporary resource management is doubly burdened by high rates of organic material disposal in landfills, generating potent greenhouse gases (GHG), and globally degraded soils, which threaten future food security. Expansion of composting can provide a resilient alternative, by avoiding landfill GHG emissions, returning valuable nutrients to the soil to ensure continued agricultural production, and sequestering carbon while supporting local communities. Recognizing this opportunity, California has set ambitious organics diversion targets in the Short-Lived Climate Pollutant Law (SB1383) which will require significant increases (5 to 8 million tonnes per year) in organic material processing capacity. This paper develops a spatial optimization model to consider how to handle this flow of additional material while achieving myriad social and ecological benefits through compost production. We consider community-based and on-farm facilities alongside centralized, large-scale infrastructure to explore decentralized and diversified alternative futures of composting infrastructure in the state of California. We find using a diversity of facilities would provide opportunity for cost savings while achieving significant emissions reductions of approximately 3.4 ± 1 MMT CO_2_e and demonstrate that it is possible to incorporate community protection into compost infrastructure planning while meeting economic and environmental objectives.

## Abstract 2

This policy analysis provides a framework and methodology for policymakers and stakeholders to incorporate environmental, economic, and social objectives into compost facility planning to support broader organic waste and climate change mitigation goals.

## 1 Introduction

Increasing the use of composting is frequently touted as an opportunity to simultaneously divert organic materials from landfills, where they produce high quantities of methane, and to sequester carbon (C) in degraded soils, offering a double win for climate change mitigation.^1−4^ To help ensure a just sustainable transition, composting also offers an opportunity to unite climate change mitigation and landscape restoration goals with broader social justice objectives.^5,6^ In this paper, we evaluate overlapping economic and socio-environmental goals associated with developing new composting infrastructure in the state of California.

California has implemented a suite of programs and policies to tackle landfill emissions, the source of 20% of the state’s anthropogenic methane emissions.^7^ The most comprehensive piece of legislation is Senate Bill 1383, passed in 2016, (Short-lived Climate Pollutants (SLCP): Organic Waste Reductions; Lara, Chapter 395), which sets bold goals for landfill methane reduction, including a 75% reduction in the statewide disposal of organic waste from the 2014 level by 2025.^8^ Much of this material is expected to be composted, but at present there is not enough capacity to handle all of this diverted organic waste. A key objective of this bill is to prompt a paradigm shift in how waste is managed in California, creating a circular system in which food scraps and organic materials are recovered and repurposed, rather than a linear system in which they are landfill-bound. This transformation requires new infrastructure and significant changes in the management of organic materials, including how they are used, collected, processed, and redistributed.

California is increasingly looking to its working lands (grazed grasslands and cultivated croplands) for climate mitigation and biodiversity enhancement. As part of its Natural and Working Land Climate Smart Strategy and the Healthy Soils Program, the state has set clear goals around increasing carbon (C) storage and improving soil health through compost application.^9,10^ Once land applied, compost, the product of controlled aerobic microbial decomposition, promotes C sequestration in soil by stimulating microorganism activity and increasing the soil’s photosynthetically derived C biomass.^11−14^ Compost can increase water retention and nutrient availability, and can displace the use of traditional fertilizers.^15^ California has over 24 million acres of rangelands and cultivated cropland which make up nearly a fifth of the State’s land area—they provide a significant compost application opportunity.^16,17^

Enhancing the role of compost in California’s waste management paradigm also offers an opportunity to incorporate considerations of environmental and economic justice into the state’s sustainability transition. Traditional waste management has historically burdened disadvantaged communities with disproportional environmental harms;^18^ repeated studies have shown waste facilities tend to be concentrated in areas of relative social deprivation.^19^ Beyond methane emissions reductions, composting can benefit food security and economic resiliency through local production of food and expanded employment opportunities, especially when developed at smaller, community-centered scales.^20^ Actively considering the social and economic cobenefits of climate change mitigation policies such as organic waste diversion can offer benefits to disadvantaged communities in line with California’s codified environmental justice (EJ) principles.^21^

This is the first study that we know of to focus on the necessary expansion of composting infrastructure in California and to consider the diversity of facility types available to meet organics management capacity needs and social justice goals. Building on field trials that demonstrate the carbon (C) sequestration potential of a single application of compost,^22,23^ past work has quantified the potential costs and emissions benefits associated with compost diversion in California.^24−27^ However, these studies focus only on existing conventional, industrial-scale composting facilities and do not consider the variety of composting facility types that may be deployed to simultaneously meet both environmental and social objectives.^28^

To address this knowledge gap, we investigate how California’s additional composting infrastructure needs can be met under a range of social, economic, and environmental constraints. We build on past studies analyzing existing infrastructure using a similar lifecycle approach^24,25,28^ to address three primary goals: (1) review the variety of composting facility types and their different technical and economic factors; (2) estimate the distribution and size of new composting facilities needed to minimize cost or emissions; and (3) evaluate whether social and environmental policy objectives can be met by incorporating community and on-farm composters. Although this analysis centers on California, the results are intended to provide a framework and methodology for decision-makers across the country.

## 2 Materials and Methods

This analysis was completed in three steps. First, we collected spatially explicit data estimates on available biomass feedstocks across the state, existing composting capacity, land-use characteristics for compost application on working lands, and relevant policy considerations for composting infrastructure development, including air quality and contemporary pollution burden. Second, we consulted literature and expert stakeholders to develop a typology of composter facilities, in which small-scale community-based and on-farm composting were considered alongside conventional centralized, large-scale infrastructure. This allowed for a better comparison of trade-offs based on bundled attributes of each facility type and for appropriate parametrization of our model. Finally, we constructed a linear optimization model to evaluate these variables and determine where best to locate new composting facilities under policy-relevant objectives and constraints.

We developed a set of 12 scenarios that minimized either cost or GHG emissions to robustly assess the impact of incorporating facility diversity and socio-environmental considerations in new compost infrastructure development. The results of the model return least-cost supply chains for compost, including geographically bounded estimates of new build per facility type (in tonnes), mass and distance of feedstock and compost moved, net CO_2_e reductions and cost. Modeled scenario results outline a framework for organic material management that incorporates the social and ecological objectives of California’s climate change mitigation, working land, and environmental justice planning.

### 2.1 Compost Facility Considerations

To achieve its compost-related diversion, soil health, and mitigation goals, the State of California will need to add 5–8 million tonnes/year of additional compost capacity to handle municipal solid waste material newly diverted away from landfills.^29,30^ The distribution and size of these new facilities will depend on regional attributes, like land use in the area, feedstock availability, end-market access, and air quality and community health considerations. Though it possesses numerous beneficial qualities, compost is an expensive product to transport, as it is water-rich and heavy.^25^ As a result, the strategic placement of new facilities such that they are close to feedstock collection points and end uses is paramount to minimize costs and emissions for the whole system.

The analysis considered two feedstock source types: the organic fraction of municipal solid waste (OFMSW, hereafter MSW, consisting of food scraps and green waste) and agricultural materials (manure and orchard residue). We focused primarily on MSW in line with SB1383 goals. However, agricultural feedstocks represent an impressive volume of organic materials in the state and are included in a few scenarios to assess their potential impact on emission reduction outcomes and to envision a future where feedstocks are managed holistically across California.

We also considered compost facility heterogeneity; operations range from small-scale community facilities to on-farm agriculturally based facilities to large-scale industrial facilities, each associated with different sizes, costs, land uses, technologies, and community impacts.^20^ For example, diversified and decentralized composting infrastructure, as opposed to large industrial facilities, has been shown to reduce transportation cost, provide for greater community-buy in, and can be more resilient and adaptable to local conditions.^6,20^

Finally, we included air quality and community health considerations in compost facility development. Regional air quality constraints may restrict the type of facilities that can be developed in an area—air districts that have already reached maximum capacity for air pollutant concentrations may not be permitted to build new large industrial facilities due to the volatile organic compounds they release.^31^ For the same reason, areas that have been identified as Disadvantaged Communities (DAC) based on their high CalEnviroScreen score, which indicates that they already experience a high pollution burden, may not be able to accommodate a large industrial facility.^32^ Consideration of environmental justice is critical as the state expands composting capacity—at present, over 35% of existing composters are in DAC census blocks according to our analysis of CalRecycle data. By volume, over half of existing capacity is located in these communities. We investigated how these socio-environmental constraints can be managed while still meeting state capacity needs.

#### 2.1.1 Facility Typology

**Figure 1:**
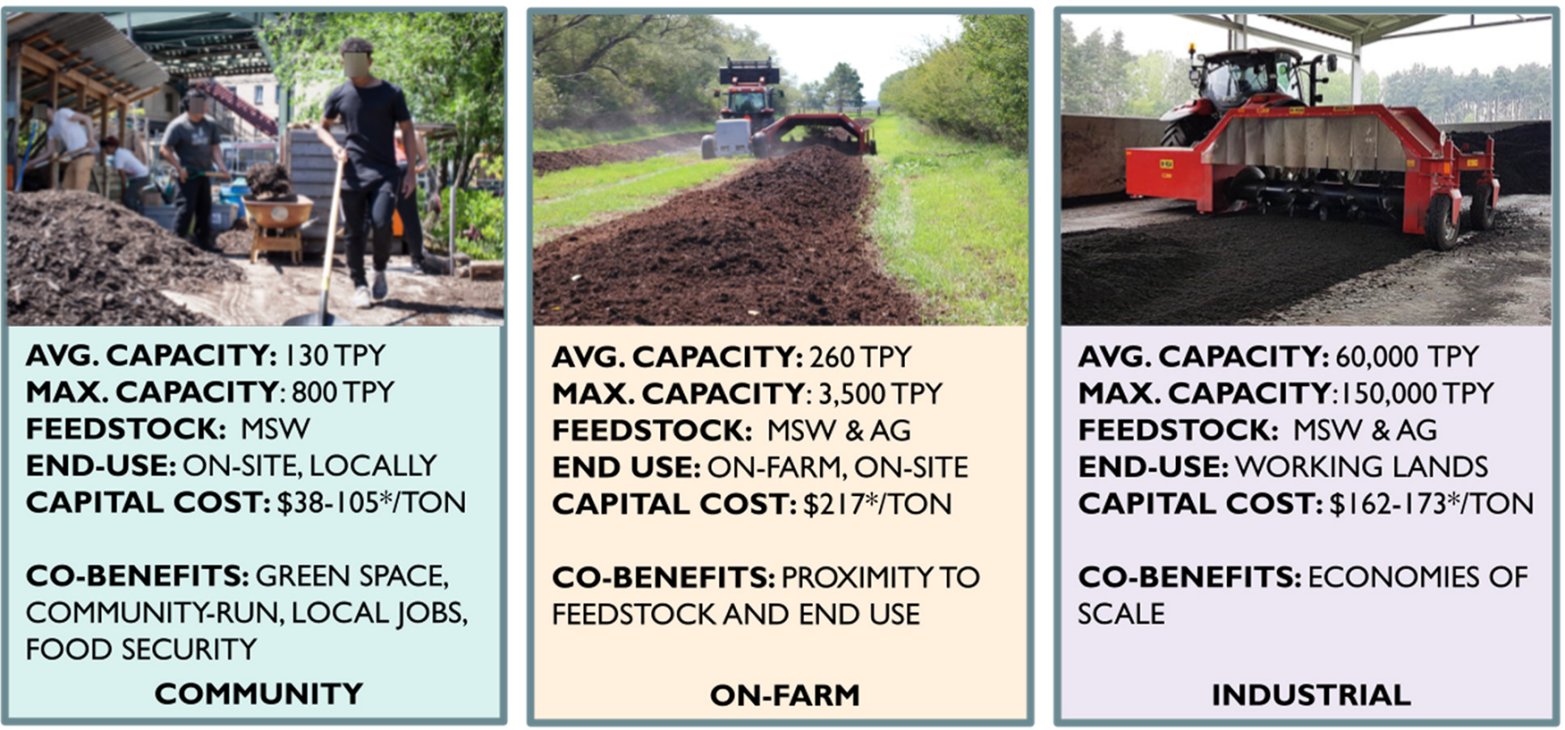
Composting facility typology. Capacity is measured in tons per year (TPY) (tonnes). Feedstocks include municipal solid waste (MSW) and agricultural residue (AG). Capital costs include a per ton estimate for construction of a new facility—land costs and labor are not included. *Denotes values used in the analysis.

Figure 1 illustrates a general compost facility typology drawn from expert interviews and literature review. We highlight several key differentiating factors for facility type including size, feedstock, end-markets, land use type, and capital costs. Maximum facility size, feedstocks, and capital costs were all included in the next step of the analysis, the spatial optimization model. It is important to note that facilities exhibited important differences in community benefits, though these are challenging to quantify and were not directly modeled in this work.

**Figure 2:**
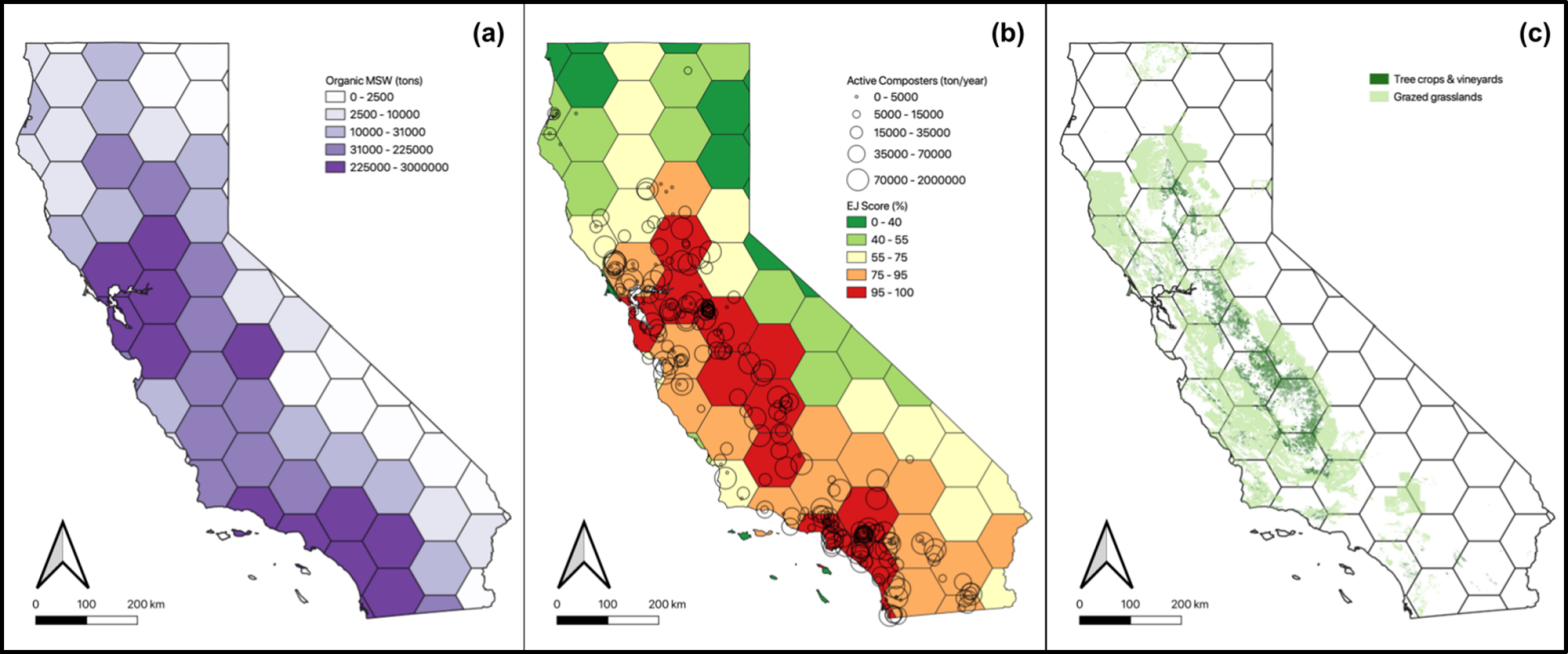
Key model inputs and zones. Black hexagons delineate the zones used in this analysis. Panel (a) presents organic fraction of municipal solid waste (MSW) within each zone. Panel (b) shows both the locations of existing composters and the maximum CalEnviroScreen Environmental Justice score in each zone. Panel (c) displays the distribution of end-markets for land application of compost.

Industrial composters are currently the dominant model of compost processing facilities in the state. The average facility processes 60 000 tonnes per year of capacity.^33^ Existing facilities are located throughout the state, with a noticeably heavy presence in the Central Valley, and near urban centers in Northern and Southern California (see Figure 2). They can process most feedstocks (including MSW, agricultural, and wood) and are associated with high capital costs and stringent permitting requirements based on throughput and footprint.

On-farm composters are those facilities located on actively operating agricultural enterprises. They tend to be smaller in scale, and to process uncontaminated agricultural residue (like manure, crop, and orchard residue) as feedstocks, though can accept MSW as well.^34^ Finally, community composters tend to be associated with community gardens and operated by nonprofit organizations to produce soil amendments for local community members. They are the smallest type of compost facility and often provide a range of social cobenefits, including added community green space, education opportunities, local jobs, food security, and embeddedness in communities. Although community composters produce much less compost than industrial processors and face unique challenges related to land access, feedstock contamination, and processing capacity, there is increasing interest in using these decentralized facilities across the state and beyond as demonstrated by recently established sources of funding.^5,6^ We investigate the technical and economic potential of on-farm and community composters given their smaller throughput capacity.

### 2.2 Siting Model

Given this facility typology, a linear programming model was developed to estimate build-out of compost infrastructure across available facility types and efficiently allocate newly diverted organic feedstocks from generation through end-use land application. We developed two functional forms for this model, one in which the objective is to minimize cost and one in which the objective is to minimize emissions. This spatially explicit model was used to evaluate multiple scenarios under a series of physical and policy-motivated constraints.

To effectively model the heterogeneity of resources across California, we regionalized the state into 78 hexagonal zones shown in Figure 2, which allow for more granularity and consistency than a county-scaled model. Although the zones are not uniform given the geography of the state, they reflect realistic waste management and compost market regions. The use of hexagons aligns our zones with the scale and radii of average collection and hauling distances for a robust assessment of transportation logistics and resource distribution.

Each zone was associated with an array of attributes relevant to the decision of whether to site a new composting facility: these included land use, feedstock availability, existing composting infrastructure, end-market access (i.e., cultivated cropland or rangeland where finished compost can be applied), air quality indices, and environmental justice scores. The solved results of the model are the capacities (in tonnes) of each newly built facility type per zone. More information on model formulation, parameter descriptions and data sources can be found in the Supporting Information (SI).

A set of scenarios were developed to reflect potential policy objectives, detailed in Table 1. Business-as-usual (“BAU”) was defined by limiting potential builds to industrial only, both with and without a maximum cap allowed per zone. “Baseline” incorporated the additional facility types of community composters and on-farm composters. The “Land-use” scenario incentivized community and/or on-farm facilities to align land use and facility characteristics by limiting the amount of industrial facilities built in zones where the proportion of land classified as “developed” or “agricultural” was above the 60^th^ percentile across all zones. Under this scenario, in those zones, maximum industrial capacity was uniformly reduced by 75%. Maximum community was increased 100% in urban zones; on-farm capacity was increased 100% in agricultural zones. The “EJ” scenario prohibited building industrial facilities in DAC zones, defined as those with EJ scores above 75%.^32^ Another scenario (“AQ”) also prohibited industrial facilities in zones that included any portion of an impacted airshed. These two constraints were combined in the “EJ & AQ” scenario. A final scenario did not allow industrial facility build in any zone (“Community & On-Farm”).

**Table 1:** Scenario Definitions for Each of 24 Scenarios

scenario name	feedstock	objective	constraints
BAU No Cap	MSW	cost; emis.	only industrial, no cap on build per zone
BAU Cap	MSW	cost; emis.	only industrial, cap on build per zone
Baseline	MSW	cost; emis.	facility diversity (all types permitted)
Land-use	MSW	cost; emis.	incentives for community and on-farm by zone land-use
EJ	MSW	cost; emis.	no industrial in EJ zones
AQ	MSW	cost; emis.	no industrial in AQ zones
EJ & AQ	MSW	cost; emis.	no industrial in EJ or AQ zones
Comm. & On Farm	MSW	cost; emis.	no industrial allowed
BAU Cap +	MSW + AR	cost; emis.	both feedstocks; only industrial
Baseline +	MSW + AR	cost; emis.	both feedstocks; facility diversity
Land-use +	MSW + AR	cost; emis.	both feedstocks; land-use incentives
EJ & AQ +	MSW + AR	cost; emis.	both feedstocks; no industrial in EJ & AQ zones

We also evaluated four of these scenarios (“BAU Cap”, “Baseline”, “Land-use”, “EJ & AQ”) with both the organic fraction of MSW and agricultural residue, for a total of 12 scenarios, each of which was evaluated under both cost and emissions minimization objectives. For both objective functions, the solved model returns the tonnage of new composting-facility builds of each type per zone, the total cost (USD), total CO_2_e emissions reductions, and the amount and distance (tonne-km) of organic materials moved in each scenario. Emissions estimates represent a combination of annual avoided emissions from diverting organic feedstocks away from landfills, emissions associated with transporting materials and producing compost, and the county-specific annual net carbon sequestration benefit from a one-time compost application to crop or rangelands.^35^ Results present annual values for material handling and one-time compost application.

We used the open-source package, CVXPY to solve the optimization problem with an academic license of the GUROBI software. This package is used for a variety of mathematical programming problems, including linear programming, quadratic programming, and mixed integer linear programming among others. We rely on their linear programming solutions. The model is implemented in the Python programming language.

### 2.3 Sensitivity Analysis

The parameters used in this model are displayed in SI Table S1 and are consistent with the existing literature evaluating the techno-economic potential of compost applications. A parametric sensitivity analysis was performed to evaluate the impact of five key input uncertainties on the results through 15 additional scenarios, presented in Table S3 in the SI.

Overall costs and emissions are both a function of organic material flow patterns, feedstock to compost conversion rate, compost processing emissions, diversion rates, and upfront capital costs for facility development. First, we evaluated the impact of feedstock constraints as defined in our typology. In this sensitivity analysis, on-farm only accepted agricultural residue while community composters accepted both municipal and agricultural feedstocks. Industrial facilities always accepted all feedstocks.

The impact of volumetric conversion during composting was assessed at low (50%) and high (150%) levels of the initial parameter. We also include a higher estimate of the greenhouse gas emissions associated with producing compost (or processing emissions) drawn from the literature, as well as lower estimate of on-farm capital cost. Finally, while the focus of our assessment was on compost, as part of the sensitivity analysis we include one scenario that alters diversion rates to include the potential role of anaerobic digestion (AD) in absorbing a portion (40%) of the diverted organic material as suggested by SB1383.^36^ This scenario also addresses potential changes in agricultural feedstock availability due to changing industry dynamics.

## 3 Results

### 3.1 Technical Build

**Figure 3:**
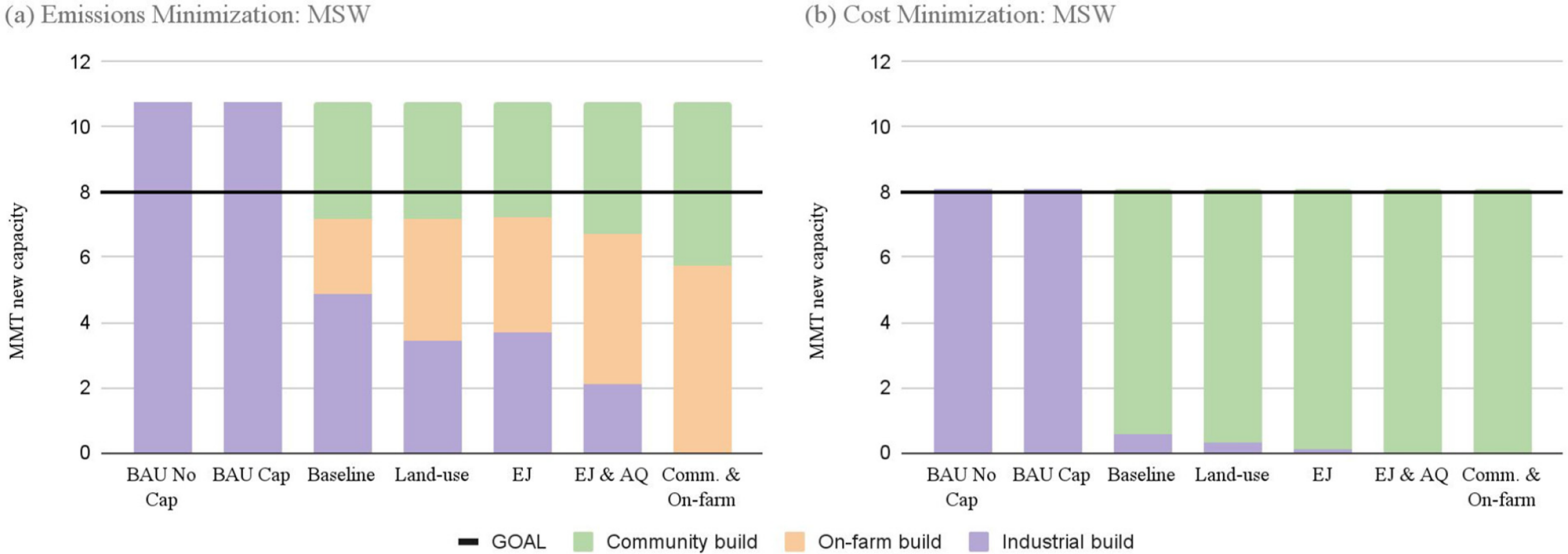
Facility build by composter type for MSW only scenarios. The horizontal axes denote the scenario and the stacked bars represent new capacity built in that scenario. Panel (a) shows results for the emissions minimization runs and panel (b) shows results from the cost minimization runs. The black line indicates the amount of new composting capacity necessary to meet SB1383 goals.

**Figure 4:**
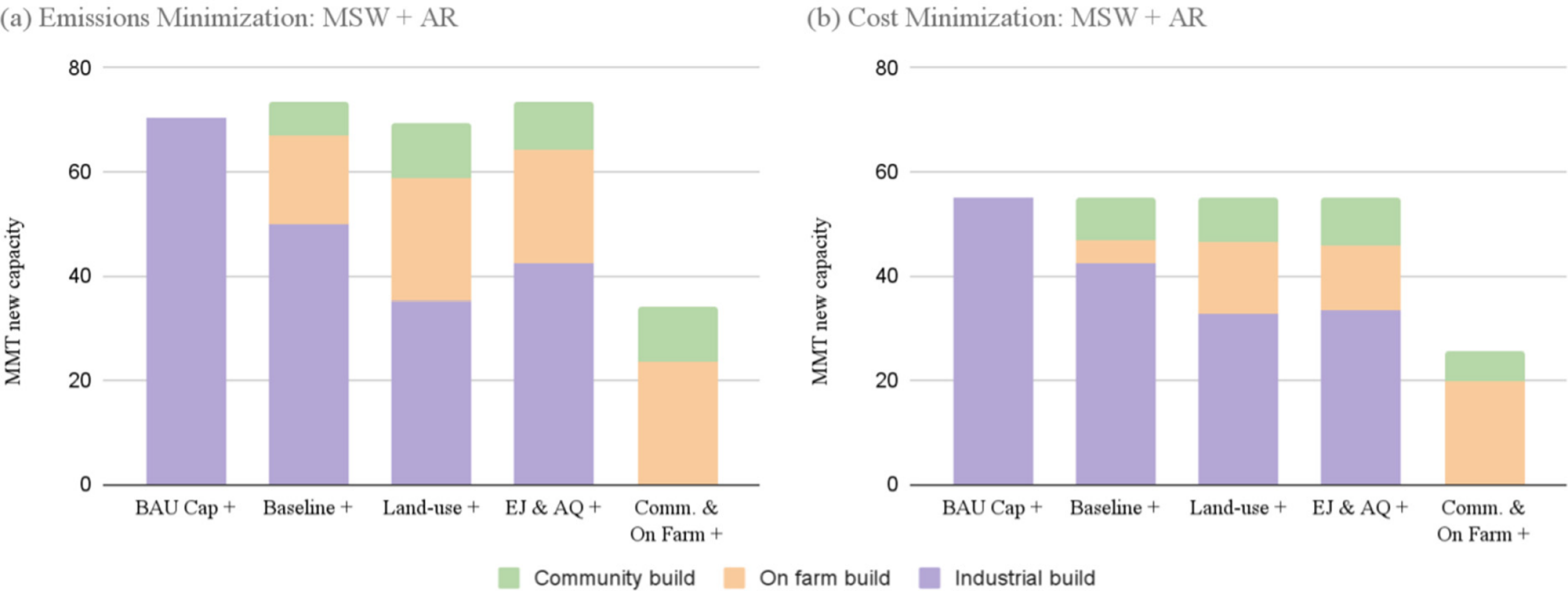
Facility build by composter type in scenarios that account for MSW and agricultural residue. The horizontal axes denote the scenario and the stacked bars represent new capacity built in that scenario. Panel (a) shows results for the emissions minimization runs and panel (b) shows results from the cost minimization runs.

The results for both cost and emissions objectives demonstrate that incorporating facility diversity and environmental justice considerations can cost less than BAU scenarios, on both a per tonne and overall basis, while resulting in approximately similar levels of emissions reductions (Tables 2 and 3). GHG reductions include both the C sequestration benefits of using compost as a soil amendment and the avoided emissions from diverting organic waste from landfills with the latter constituting the bulk of the emissions reductions modeled here. Under a cost minimization objective there is less new capacity built overall (Figures 3 and 4), with each MSW scenario just meeting the SB1383 diversion requirements of 8 MMT of new capacity, leading to fewer opportunities to sequester carbon with newly processed compost. No on-farm composters were constructed in these scenarios, likely due to the high up-front capital costs.

**Table 2:** Cost Minimization Model Results for Main Scenarios

scenario name	GHG reduction (MMT CO_2_e/yr)	cost (million USD)	USD/tCO_2_e	USD/t of new capacity	mass distance (10^6^ t-km)
BAU No Cap	2.5585	$1587	$620.25	$196.25	326
BAU Cap	2.6112	$1532	$586.52	$189.40	100
Baseline	2.4685	$906	$367.16	$112.08	514
Land-use	2.4489	$903	$368.71	$111.67	572
EJ	2.4327	$900	$369.90	$111.28	634
AQ	2.4278	$898	$369.76	$111.02	655
EJ & AQ	2.4281	$898	$369.76	$111.02	655
Comm. & On Farm	2.4285	$898	$369.66	$111.02	655
BAU Cap +	15.8297	$12 157	$768.01	$220.54	6324
Baseline +	16.4445	$10 965	$666.76	$198.90	4662
Land-use +	15.2604	$12 383	$811.42	$224.62	7855
EJ & AQ +	15.3223	$12 412	$810.06	$225.16	8006

**Table 3:** Emissions Minimization Model Results for Main Scenarios

scenario name	GHG reduction (MMT CO_2_e/yr)	cost (million USD)	USD/tCO_2_e	USD/t of new capacity	mass distance (10^6^ t-km)
BAU No Cap	3.3824	$2164	$639.75	$200.70	587
BAU Cap	3.4661	$2069	$596.82	$191.87	240
Baseline	3.4950	$1776	$508.21	$164.74	112
Land-use	3.4456	$1896	$550.38	$175.89	332
EJ	3.4606	$1812	$543.89	$174.57	261
AQ	3.4348	$1873	$545.35	$173.74	373
EJ & AQ	3.4348	$1906	$554.76	$176.74	373
Comm. & On Farm	3.4025	$1896	$557.10	$175.81	497
BAU Cap +	19.9981	$16 692	$834.69	$236.59	11,791
Baseline +	22.0964	$16 047	$726.24	$218.33	7,827
Land-use +	19.3146	$16 740	$866.69	$241.70	12 686
EJ & AQ +	20.1838	$18 210	$902.23	$247.76	14 652

As additional constraints were included in the modeled scenarios, emissions reductions decreased slightly, while cost per ton of CO_2_-equivalent avoided also decreased. This result is consistent across both cost and emissions minimizations objectives and both feedstock scenarios. Overall cost associated with transporting feedstocks, constructing facilities, and land-applying compost dropped significantly, even as the hauling and collecting distance increased. This is especially pronounced in the MSW feedstock-only runs: BAU costs are about 50% higher than scenarios that allow diverse facility types to be built. One unexpected result was that the AQ and EJ & AQ scenarios yielded identical results from the optimization model under the cost minimization objective. We display both results in Tables 2 and 3, but only present the mapped distribution of new facilities for EJ & AQ in Figure S2.

In the cost minimization model runs, on-farm composting facilities are only built in the scenarios that include both the organic fraction of MSW and agricultural feedstocks (the scenarios marked with a “+”). In the emissions minimization runs, this facility type was utilized even under the MSW scenarios to absorb organic material around major population centers that are surrounded by agricultural landscapes, decreasing the transport distance (t-km) that material needed to be moved.

Adding agricultural feedstocks into the model considerably increased both the cost and the net C sequestration approximately 6-fold due to the increased volume of organic matter that could be processed into compost and land applied. Both cost per ton of new capacity and cost per ton of CO_2_e avoided and sequestered was nearly doubled in these scenarios. The scenarios that include MSW and agricultural feedstocks highlight the contribution of large scale, industrial composting facilities in organic waste management—community composters and on-farm composters alone are not able to handle the volume of material produced.

In general, the spatial distribution of new composters tended to cluster around the large population areas in the southern coast and Bay Area (see SI for maps). Cost minimization runs restricted to MSW feedstocks privileged community composters, resulting in a similar spatial arrangement across scenarios as constraints are added to the baseline runs (Figure S2). Under the emissions minimization objective we found greater variability across scenarios, including limiting industrial composting capacity in the Central Valley where EJ scores tend to be higher and incorporating more on-farm composting in the southern and Bay regions (Figure S3). When both agricultural and MSW feedstocks were included, this spatial trend inhibiting industrial composters in the Central Valley is even more pronounced (Figure S4).

The spatial patterning of the land use scenario, in which the maximum capacity for community composters and for on-farm facilities was increased in urban and agriculturally dense zones, respectively, and the maximum capacity of industrial facilities was lowered, was strikingly similar to the results of the baseline scenario in which all three facility types could be used. This is likely driven by the existing spatial arrangement of feedstock sources from municipal areas and working lands for compost application as well as the high cost (both in dollars and greenhouse gas emissions) of transporting compost large distances. The inclusion of EJ and air quality constraints had a more noticeable impact on the distribution of new composters by restricting industrial facilities in the Central Valley and South Coast regions. Surprisingly, this scenario still results in a high level of industrial facility build out by shifting development of industrial composters to the northern regions of the state, particularly under the emissions minimization run (Figure S5).

### 3.2 Sensitivity Analysis Results

The results of the sensitivity analysis demonstrate the influence of select key parameters on overall climate change mitigation impact and associated cost (Table S3). Relative to the Baseline scenario, altering the conversion rate of waste to compost alters the overall mitigation and cost, but, unsurprisingly, does not change build volumes. In the scenario where diversion rates were lower, only community composting facilities were built. Using a higher estimate of composting processing emissions had little effect on CO_2_e emissions reductions, but resulted in less on-farm capacity and more industrial.

Altering the allowed feedstocks accepted at composter types had little effect on the land-use, EJ, and EJ & AQ scenarios, except to slightly increase the amount of community composting built and decrease the other two facility types. This result is unsurprising given that community composting is the lowest cost and faces the least constraints under those scenario runs.

One surprising result from the sensitivity analysis was the impact of lowering the on-farm capital cost to the lower literature estimate. In this run, the cost per ton of carbon sequestered was much higher than in Baseline for MSW feedstock because the model builds a much greater volume of on-farm facilities to achieve approximately similar emissions reductions, but the distance materials must be moved is significantly higher, which drives up the overall cost. This suggests that transportation costs likely drive cost outcomes.

## 4 Discussion

We have evaluated the techno-economic potential of expanding compost infrastructure as a cost-effective climate change mitigation approach that complements healthy soil initiatives and just transition goals. The overall results of this analysis demonstrate that incorporating social environmental justice objectives into waste management planning can be accomplished without sacrificing environmental impact or increasing overall cost. We further reveal that the diversification of composting facility types and feedstocks allows policymakers to not only better meet capacity needs, but also provides the opportunity to reduce costs and maximize emissions reductions.

Expansion of small-scale community and on-farm composting programs may offer significant cobenefits while advancing the policy goals of SB1383 and as such it is critical to understand the technical and economic potential of incorporating them into planning processes. The current stage of targeted investment in new waste management infrastructure presents an opportunity for prioritizing EJ in sustainability transition planning.^37^ Through this high-level analysis, we aim to provide a framework for decision-makers as they consider these investments in new waste management infrastructure from a standpoint of overlapping climate change mitigation and social vulnerability.

Our findings complement and uphold existing analyses that have also utilized a broad lifecycle assessment, including the cost and emissions associated with organic waste diversion through existing composting facilities and subsequent land application, to evaluate statewide nutrient cycling systems. Harrison et al., for instance, found that landfill diversion and farmland compost applications could reduce emissions by an average of 6.3 MMT CO_2_e annually.^25^ Delonge et al., focusing on compost application to grazed grasslands, demonstrated that the widespread use of compost may provide a net savings of more than 8 MMT CO_2_e over 15 years if applied to just 6% of California’s rangelands.^24^

While we found lower estimates of overall emissions reductions than previous studies, the results presented here could be considered conservative estimates for two important reasons. First, the model does not account for avoided fertilizer use on cultivated cropland, which would likely be displaced by the use of compost. Synthetic fertilizer is energy-intensive to produce. Thus, emissions reductions associated with the application of compost in lieu of traditional fertilizer may be greater than what is modeled here. Second, because this analysis was focused on siting of new infrastructure to meet the needs set out by SB1383, the goal was to present a cross-sectional analysis of scenarios that the state and local jurisdictions could reasonably pursue. Consequently, the findings reflect only a single year of facility development and emission outcomes, rather than estimates of long-term impacts. Once these facilities are built, we can assume that the operation and maintenance costs, which are typically proprietary, would be much lower than the upfront capital costs, while avoided landfill emissions and C sequestration could be expected to continue. Once these facilities are up and running, the cost per tCO_2_e abated will likely be much lower. Future work could build on this analysis to address both considerations and produce more refined estimates of long-term soil C stocks resulting from compost applications.^38^

This analysis could also be extended in the future through the inclusion of processing emissions associated with different composting management practices and feedstocks. Field research is currently underway that may enhance the differentiation among facility types in the typology developed here. Data were not available for land-use price or operating costs, which is a limiting factor for this analysis that could be addressed by future work. Changing dynamics of agriculture that limit availability of manure and residues may also be fruitfully explored in later studies, such as increased fallowing of land due to California’s Sustainable Groundwater Management Act or projected closures of dairy operations in the state.

Although this model attempts to capture some of the complexity of the regulatory process of siting and constructing new composting infrastructure through air quality regulations, structural barriers including land-use zoning can vary on a city-by-city basis and were not represented in this model.^26,39^ These barriers include convoluted and cost-prohibitive permitting processes, lack of reliable feedstock collection systems, material contamination challenges, and lack of education about composting best practices.^11^ Expanding composting infrastructure in a way that centers cobenefits for climate, land, and community will require attention to the heterogeneity of policy contexts, including concerted efforts to expand organic material collection programs across the state and create central repositories for data on where these programs exist.

Across the country, contemporary waste management paradigms are characterized by high rates of organic material disposal in landfills, contributing 16% of anthropogenic emissions of the potent greenhouse gas methane,^1,40^ and perpetuation of historical harms from pollution on disadvantaged communities.^18^ At the same time, soils continue to lose C due to conventional agricultural practices.^3,41^ Engineering approaches and nature-based solutions to climate change mitigation policies can offer remediation opportunities on each of these fronts through thoughtful, participatory planning and development of new infrastructure. In developing a resilient and just organic waste management system, policymakers in every state should consider multiple scales of composting infrastructure to achieve benefits that incorporate cost, emissions, and community impact.

## Supporting Information Available

Full formulation, parameter values, and data sources used in the spatial optimization siting model, along with scenario maps displaying the geographical distribution of new facility capacity built by composter type (PDF)

## Abbreviations

CDFA: California Department of Food and Agriculture
GHG: Greenhouse gas
MSW: Municipal Solid Waste
