# Supplementary material for: Socio-environmental Opportunities for Organic Material Management in California’s Sustainability Transition
Source: Environ Sci Technol. 2024 May 16;58(21):9031–9. doi: 10.1021/acs.est.3c10711 (PMC11137869; doi:10.1021/acs.est.3c10711)
Supplement: Supplementary file 1 — es3c10711_si_001.pdf [file es3c10711_si_001.pdf]

SUPPORTING INFORMATION

Socio-environmental opportunities for organic material management in California's sustainability transition

AUTHOR NAMES:
Anaya L Hall*[a]
Aleksandra I Ponomareva[a]
Margaret S Torn[a,b]
Matthew D Potts[c,d]

AUTHOR AFFILIATIONS:
[a] Energy & Resources Group
University of California – Berkeley
345 Giannini Hall
Berkeley, CA 94720
United States

[b] Climate Sciences Department
Lawrence Berkeley National Laboratory
Berkeley, CA 94720
United States

[c] Department of Environmental Science, Policy, and Management
University of California – Berkeley
130 Mulford Hall
Berkeley, CA 94720
United States

[d] Carbon Direct, Inc.
17 State Street
New York, NY 10004
United States

*Corresponding author
anayahall@berkeley.edu

Number of Pages: 14
Number of Figures: 5
Number of Tables: 2

S1.1 Data Sources

Organic materials, or feedstocks, are defined as the organic materials that will be processed in composting facilities (rather than landfills). MSW feedstocks were provided at the census-tract level, while agricultural materials were assigned to the geographic county centroid. Municipal solid waste (MSW) consists of everyday items used and disposed of, ultimately ending up in the landfill. Our study was concerned with the organic fraction of MSW targeted by SB1383, which is made up of primarily food scraps and green waste (landscaping and yard trimmings) and estimated to be approximately 12 million wet tonnes of material annually[1]. We relied on 2025 food and green waste disposal rates as estimated by the 2014 CalRecycle waste characterization assessment composition values. Per SB1383, we assumed that at least 75% of this material needs to be diverted from landfill.

Although not directly targeted by SB1383, California generates organic waste from the state's vast agricultural sector in the form of manure and woody crop residue from orchards and vineyards that are currently underserved as biomass conversion facilities shut down across the state. As jurisdictions pursue additional composting infrastructure investments, it will likely prove beneficial to consider these important feedstocks as well. We relied on data from the Joint BioEnergy Institute California BioSiting website[2]. Manure data were from 2012. Orchard and vineyard residue data were projections for the year 2020. This feedstock is composed of naturally shed leaves, trimmings and prunings removed to improve plant yield, and tree removals. In line with previous studies, we assumed that 20% of total combined manure and orchard residue material will be processed in new composting facilities in order to replace the current volume of manure being applied to land as slurry[3].

Finished compost, or 'compost,' is the final product resulting from the composting process. This product is distinct from 'feedstock', which is the raw material, such as food scraps, livestock manure or yard trimmings used to produce the compost. We assumed that the new facilities in this study will generate a relatively homogenous product with a high C:N ratio (low Nitrogen) in accordance with the material characteristics of our organic feedstock inputs, which consist of a balance of nitrogen-rich substrates and carbon-rich bulking agents[4,5]. We used a consistent ratio of food waste and green waste from the MSW stream, and of manure and orchard residue from the agricultural residue stream to generate the required balance of nitrogen-rich (food scraps, manure) and carbon-rich (green waste, orchard residue) input materials to generate a balanced final compost.

Data on existing composters were obtained from the CalRecycle Solid Waste Inventory System (SWIS)[6], which records information facility size, location, status, activity category and accepted feedstock, and from CalRecycle's report "Analysis of the Progress Toward the SB 1383 Organic Waste Reduction Goals"[7], which provides summary metrics for compost facilities across the

state. There are 293 active composting facilities (or “composters”) in California (Figure 2 in main text) that currently process approximately 6 million tonnes of organic material annually, though they are permitted to accept up to 10 million tonnes. Composting operations can vary considerably in size and scale, even within the typology we describe in the main text. We use average values of throughput capacity in this model rather than land area, which often depends on local conditions[8].

We included two types of end-markets where finished compost will likely be land applied to promote soil health and carbon sequestration benefits. The first is rangelands, or grazed grasslands, which were acquired from the California Department of Conservation Farm-land Monitoring and Mapping program[9]. This dataset includes only areas where vegetation and land characteristics are suited to grazing of livestock as determined by a collaboration between the California Cattlemen’s Association and UC Cooperative Extension. We also included perennial cropland, derived from California Department of Water Resources[10]. We focused on high-value tree crops (including citrus, subtropical fruit, and nut trees and vineyards) as these are currently the primary agricultural markets for compost[1].

Different land types across the state are associated with different soil emissions and carbon sequestration rates[11]. An application rate of 4.7 dry tonnes compost/acre was used based on CDFA recommendations[12,13]. In this recommendation, application is assumed to occur once annually before planting or early in the growing season, and results in a projected annual greenhouse gas reduction/sequestration. Net sequestration rates were based on county and land type as given by the California Air Resources Board’s (CARB) use of the DeNitrification-DeComposition (DNDC) Model[12]. Land use characteristics from the National Land Cover Database were also included to allocate new composter facility types based on factors such as urban or agricultural density.

CARB Air Quality Districts were used to determine sensitive air districts that have highly restrictive air pollution threshold limits. The CalEnviroScreen dataset was used to determine the median environmental justice score for each zone, as well as whether that zone contained any census block designated as a Disadvantaged Community (DAC), as indicated by a score of 75 or above[14]. CalEnviroScreen considers a comprehensive set of environmental, health and social indicators, including pollution burden, socio-economic vulnerability, and public health concerns, to assess conditions and score census tracts across the state. These values were also used to set constraints in the model. All data-cleaning and processing was done in Python and QGIS.

SI1.2. Siting Model Formulation

Indices, objective functions, decision variables, and constraints of the spatial optimization model are detailed here.

Sets

q	Zones (1 x p)
T	Composter Types*: Industrial (IF), On-Farm (OF), Community (CC)*
i	Organic Feedstock Type: *MSW, MSW + Agricultural*
j	End-Use Type: *Grazed Grassland, Perennial Cropland*
$D_{qq'}$	Distance matrix between zone of origin and zone of new composter
$B_{qq'}$	Distance matrix between zone of new composter and zone of land application

The decision variables solved in this model are the quantity of annual throughput capacity (in tonnes) of each composter type T to build in each zone q, and how best to allocate organic materials between generating zones and land application areas. Movement of (uncomposted) organic feedstocks of different material classes are modeled separately, as are final land use types receiving finished compost. The decision variables are displayed below.

Decision Variables

$X_{q,T}$	Annual tonnage of new facility of type T in zone q
$F_{i,qq'}$	Tonnage of (uncomposted) feedstock moving between zones
$L_{qq',i}$	Tonnage of finished compost moving between zones

As noted in the main text, we constructed two objective functions to reflect policy orientations that prioritize cost or emissions. The first, minimizing cost, is a function of collection ($C_{collection}$) and hauling cost ($C_{hauling}$), which itself is dependent on distance traveled, the upfront capital required for new composting facilities (excluding land price and labor) ($C_{construction}$), and the cost to physically apply finished compost to land ($C_{spreading}$), where A_q equals the amount of finished compost applied, as defined below. $F_{i,qq'}$ is the amount of materials moving between zones into a site of new infrastructure, and $L_{qq',j}$ is the amount of material moving between zones for land application. $D_{qq'}$ and $B_{q'q}$ are the distance matrices between zones. In this function, we did not include costs from labor and land due to limited availability.

Objective Function 1: Minimize Cost

(1) $$\text{minC} = \left(C_{collection} + C_{hauling} + C_{construction} + C_{spreading}\right)$$

(2) $$C_{collection} = \text{a} \cdot \sum_{q=1}^{p} \sum_{q=1}^{p} \left(D_{qq'} \cdot F_{i,qq'}\right)$$

(3) $$C_{hauling} = d \cdot \sum_{q=1}^{p} \sum_{q=1}^{p} \left(B_{qq'} \cdot L_{qq',j}\right)$$

(4) $$C_{construction} = \sum_{q=1}^{p} \left(l_{IF} \cdot X_{q,IF} + l_{OF} \cdot X_{q,OF} + l_{CC} \cdot X_{q,CC}\right)$$

(5) $$C_{spreading} = \sum_{j} \sum_{q=1}^{p} b_j \cdot A_q$$

(6) $$A_q = \sum_{q=1}^{p} L_{qq',j}$$

The second objective function, minimizing emissions, evaluates total $kgCO_2eq$ from a solved arrangement of new composting infrastructure, including roadway collection *($E_{collection}$*) and

hauling emissions ($E_{hauling}$), annual compost processing emissions ($E_{processing}$), and emissions released during the spreading of finished compost to land ($E_{spreading}$). We subtract from these emissions the avoided greenhouse gas emissions resulting from diverting organic feedstocks away from landfill ($E_{landfill}$) as well as the expected carbon sequestration benefit of applying compost to range or cropland ($E_{sequestration}$). In these functions, we did not include fossil fuel emissions from machinery during compost processing.

Objective Function 2: Minimize Emissions

(7) $\mathrm{minE} = \left(E_{collection} + E_{hauling} + E_{processing} + E_{spreading}\right) - \left(E_{landfill} + E_{sequestration}\right)$

(8) $E_{collection} = \mathrm{r} \cdot \sum_{q=1}^{p} \sum_{q=1}^{p} \left(D_{qq'} \cdot F_{i,qq'}\right)$

(9) $E_{hauling} = \mathrm{r} \cdot \sum_{q=1}^{p} \sum_{j=1}^{p} \left(B_{qq'} \cdot L_{qq',j}\right)$

(10) $E_{processing} = \sum_{q=1}^{p} u \cdot \left(X_{q,IF} + X_{q,OF} + X_{q,CC}\right)$

(11) $E_{spreading} = \sum_{j} \sum_{q=1}^{p} w_j \cdot A_q$

(12) $E_{landfill} = \sum_{i} \sum_{q=1}^{p} \sum_{q=1}^{p} v_i \cdot F_{i,qq'}$

(13) $E_{sequestration} = \sum_{j} \sum_{q=1}^{p} s_{j,q} \cdot A_q$

The above objective functions are each subject to two kinds of constraints. First, a series of physical plausibility constraints were imposed. For instance, total build and build for each type of facility must be positive (equations 14 and 15). Further, material flow from a given zone must be less than or equal to total available organic feedstock in that zone (equation 17). Similarly, the flow into a zone is constrained by the amount of appropriate range and cropland in that zone (equation 18). The build in any given zone is limited to the flow into that zone by material type, and the total flow into new composting facilities ($F_{i,qq'}$) in each zone must be balanced by the flow out of those facilities onto land ($L_{q'q,j}$).

Constraints

(14) $0 \leq \sum_{q=1}^{t} \left(X_{q,IF} + X_{q,OF} + X_{q,CC}\right)$

(15) $X_{q,T} \geq 0$

(16) $X_{q,T} \leq T_{max}$

(17) $\sum_{T=OF,IF,CC} \left(X_{q,T}\right) \leq \sum_{q=1}^{p} O_q$

(18) $\sum_{T=OF,IF,CC} \left(X_{q,T}\right) \leq \sum_{q=1}^{p} I_q$

(19) $A_q \leq I_q$

(20) $\sum_{T=OF,IF,CC} X_{q,T} \leq \sum_{q=1}^{p} \left(F_{i,q'q}\right)$

(21) $\sum_{q=1}^{p} F_{i,qq'} = \mathrm{x} \cdot \sum_{q'=1}^{p'} \left(L_{q'q,j}\right)$

Table S1. Scalar parameter values, descriptions, and sources

Variable	Description	Value	Unit	Source
a	Collection cost (municipality to facility)	0.27	$/ton*km	*RRS (2017)*[15]
d	Hauling cost (facility to land application)	0.0875	$/ton*km	*EPA (2002)*[16]
b_j	Spreading/application cost	3.4	$/ton	*Hall (2022)*[17]
l_{IF}	Capital Cost: Industrial	173	$/ton	*Platt et al. (2014)*[18]
l_{OF}	Capital Cost: On-farm	217	$/ton	
l_C	Capital Cost: Community	105	$/ton	*Brown (2022); Platt et al. (2014)*[18]
r	Collection and Hauling Emission Factor	0.22	kgCO_2e/ton*km	*CARB (2017)*[19]
u_T	Composting Processing Emissions	57.05	kgCO_2e/ton	*Vergara & Silver (2019)*[20]*; Silver et al. (2018)*[3]
w_j	Spreading emissions	1.08	kgCO_2e/ton	*Wernet et al. (2016)*[21]
v_i	Landfill Emissions	182.9	kgCO_2e/ton	*EPA WARM* [22]
	Application rate	8	tons/acre	*Gravuer (2016)*[12]
m_{IF}	Maximum capacity per zone: Industrial	100,000	Tons per year	*Platt et al. (2014)*[18]*; SWIS (2023)*[6]
m_{OF}	Maximum capacity per zone: On-farm	35,000	Tons per year	
m_{CC}	Maximum capacity per zone: Community	25,000	Tons per year	
	Volume Change Conversion	0.58	%	*Breitenbeck and Schellinger (2004)*[23]

Table S2. Vector parameters and descriptions

**Variable**	**Description**	**Unit**
$\boldsymbol{O_q}$	Organic material generated in zone q	t
$\boldsymbol{I_{q,j}}$	Available land area suitable to compost amendment in zone q	t
$\boldsymbol{D_{qq'}}$	Distance matrix between zone of origin and zone of new composter	km
$\boldsymbol{B_{qq'}}$	Distance matrix between zone of new composter and zone of land application	km

SI1.2. Supplementary Results

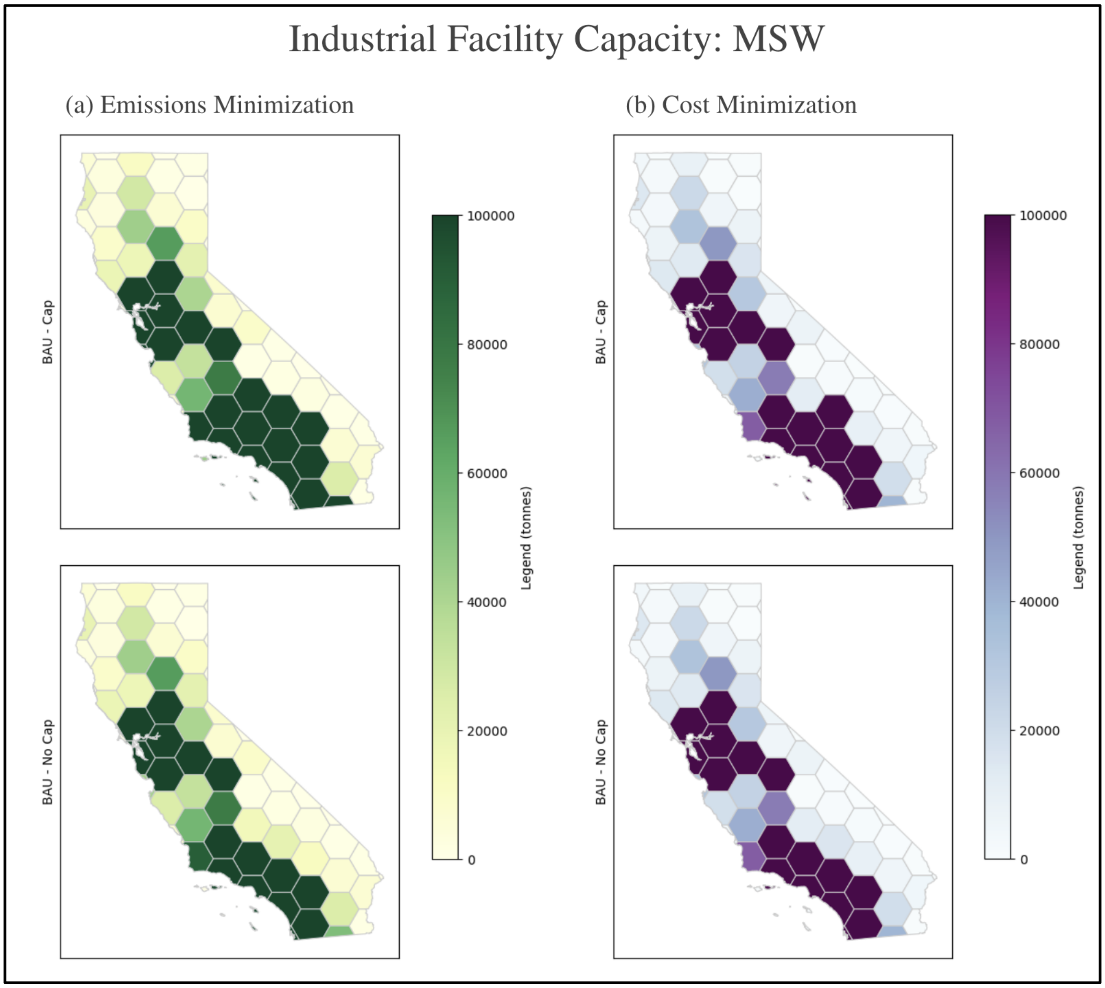

Figure S1. *Industrial Facility Capacity for scenarios where only industrial composters were allowed. Panel (a) shows the distribution of new industrial capacity under emission minimization runs. Panel (b) shows the distribution of new capacity under cost minimization runs. The second row displays results where there was no limit on industrial facilities within each zone. As a result, there tends to be higher concentrations of industrial build in the South Coast and Bay regions, surrounding population centers. When a cap is imposed, industrial facilities are distributed across more zones in those regions.*

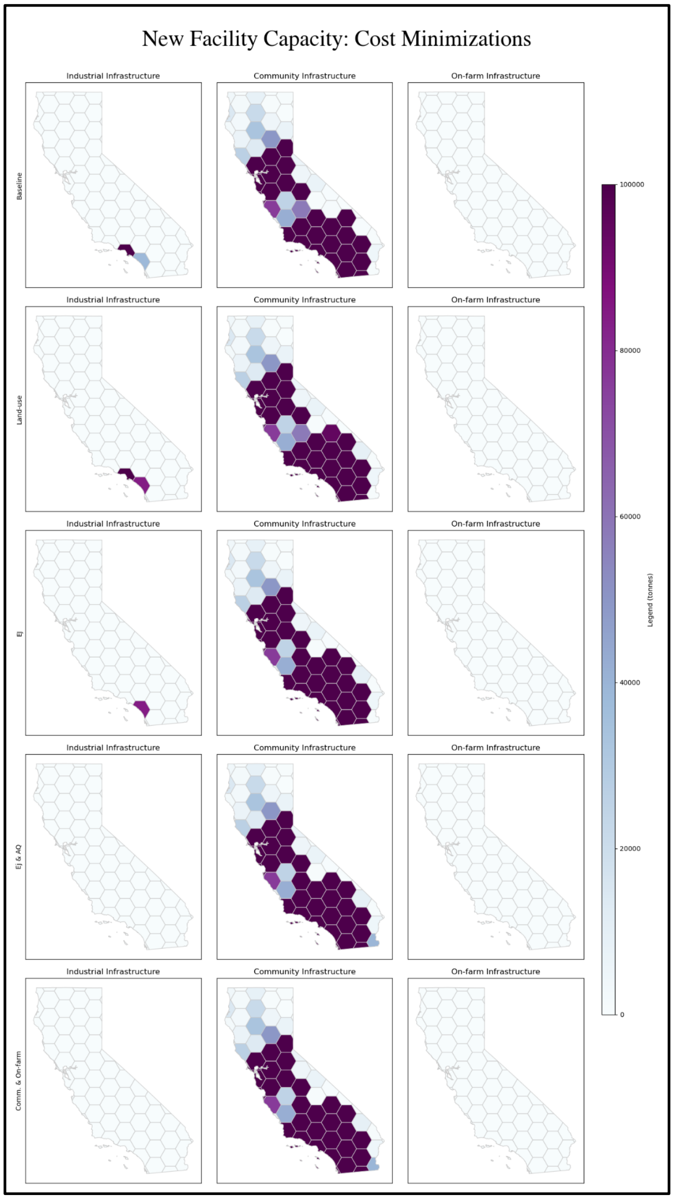

Figure S2. *Distribution of new capacity by facility type across scenarios using only MSW feedstock, under cost minimization objective*

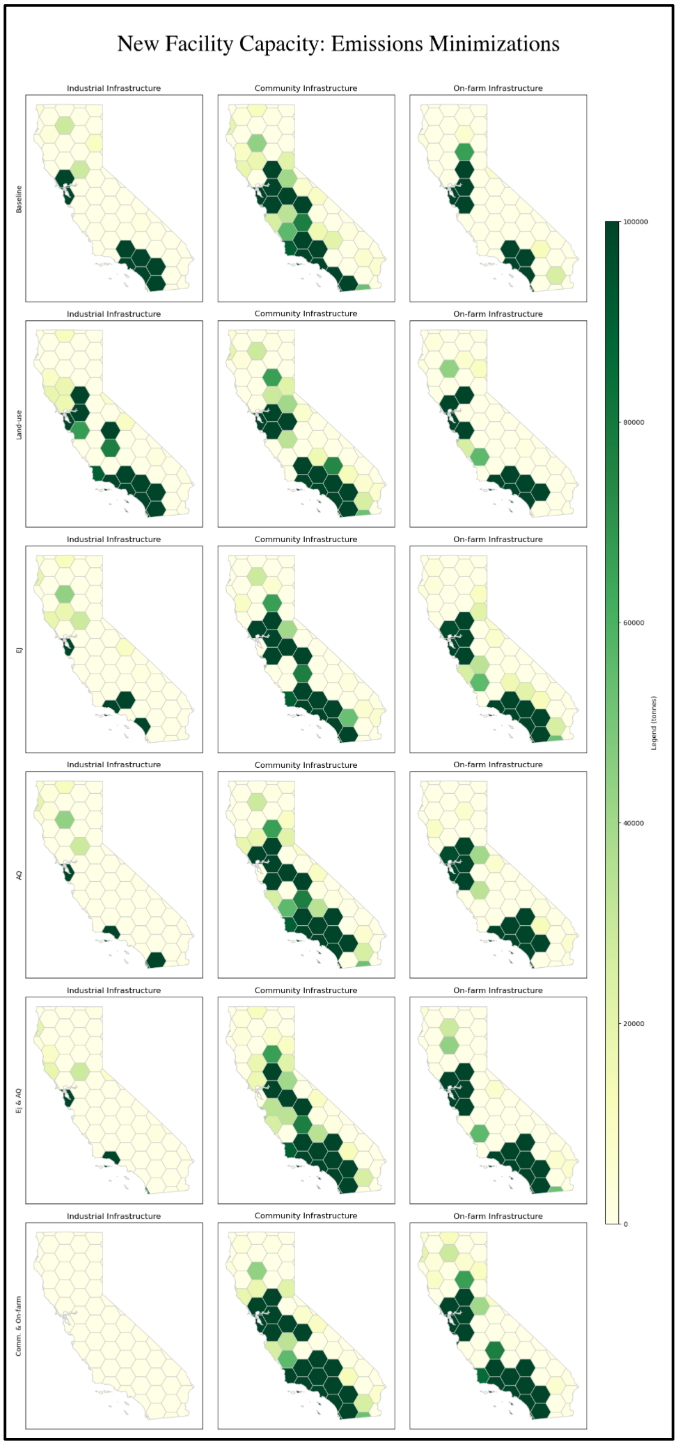

Figure S3. *Distribution of new capacity by facility type across scenarios using only MSW feedstock, under emissions minimization objective*

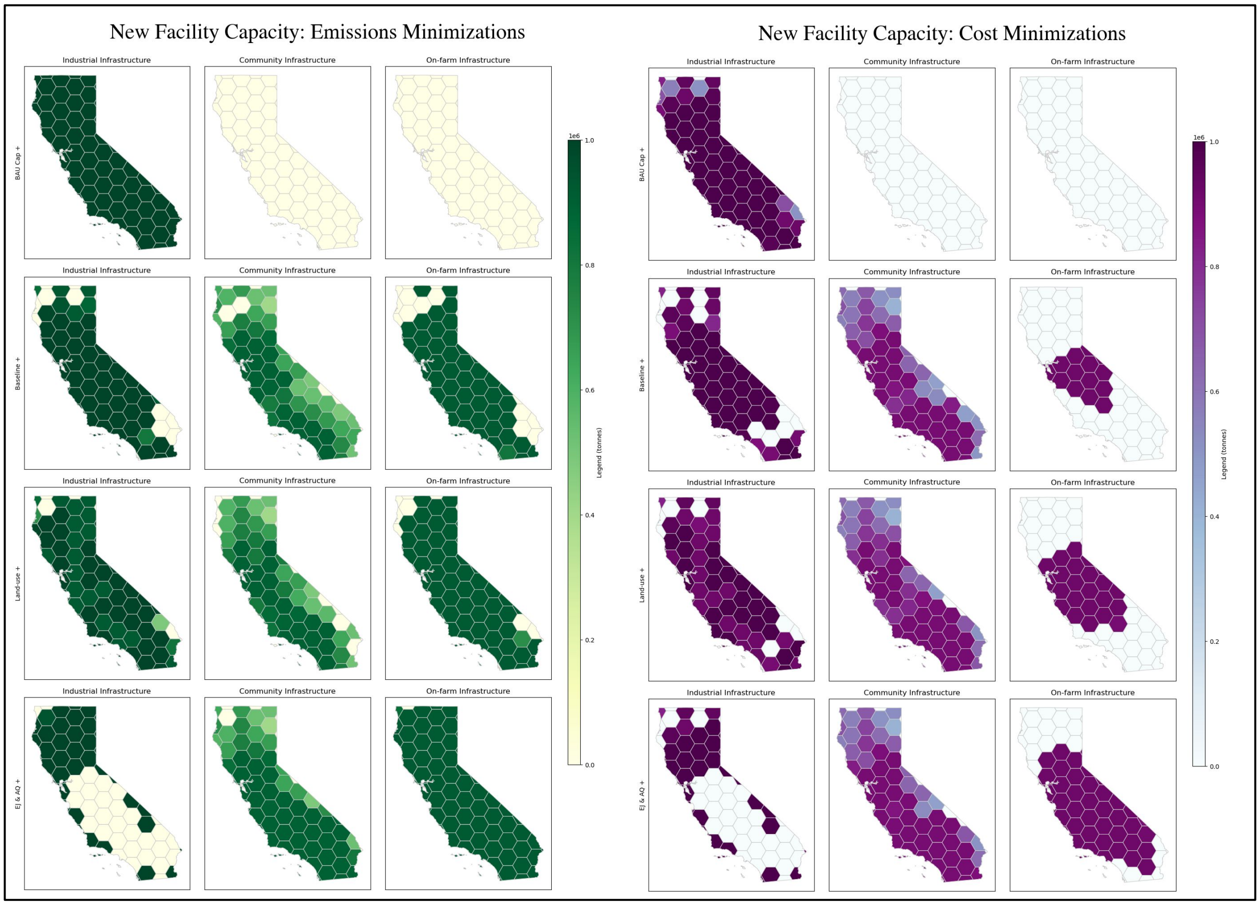

Figure S4. *Distribution of new capacity by facility type across scenarios using both MSW and agricultural feedstocks.*

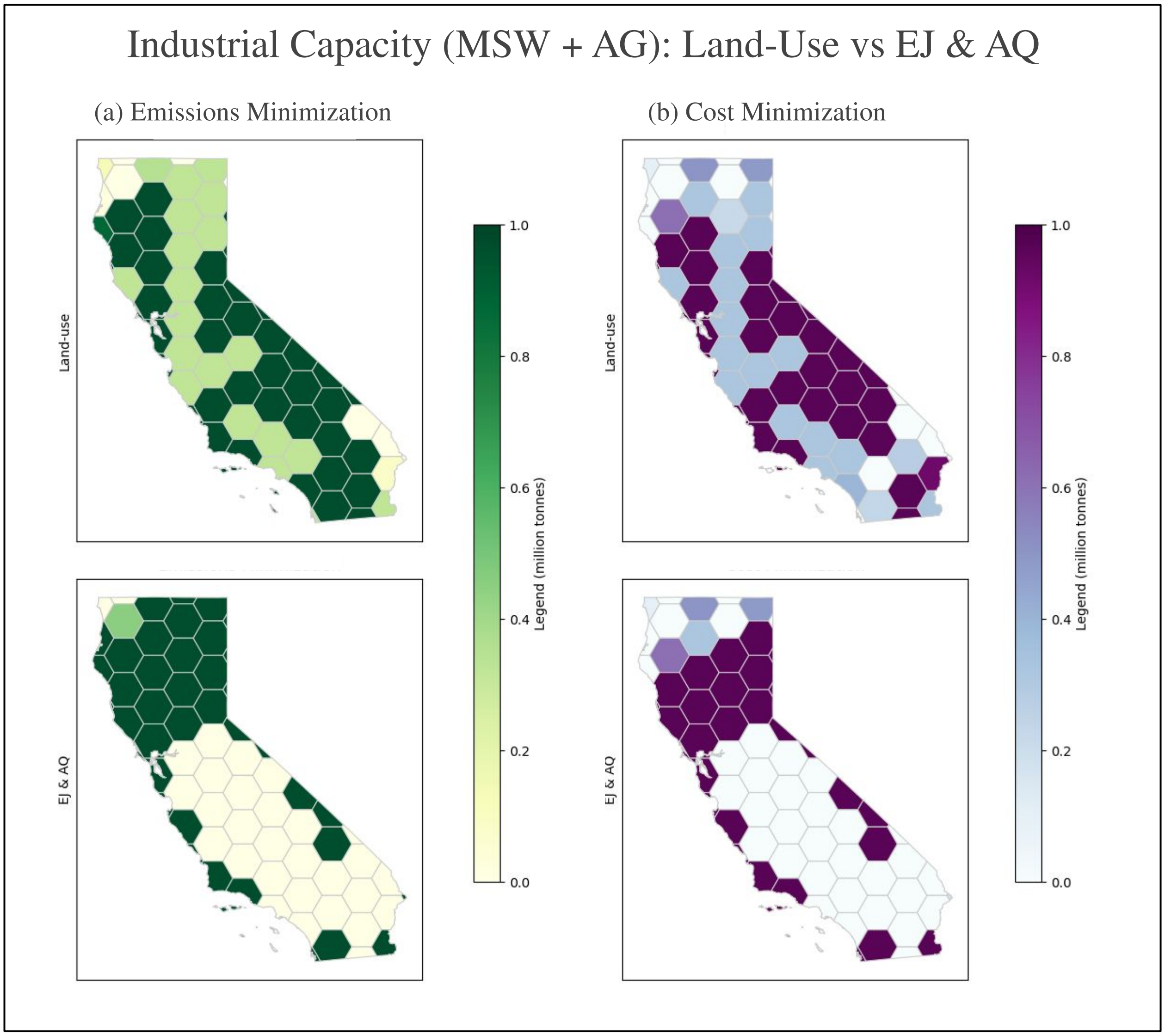

Figure S5. *Industrial facility capacity for the Land-use and EJ & AQ scenarios, highlighting the areas where industrial capacity expands or contracts under environmental justice and air quality constraints. Panel (a) shows the distribution of new industrial capacity under emission minimization runs. Panel (b) shows the distribution of new capacity under cost minimization runs.*

Table S3. Sensitivity Analysis Results

Scenario Name	Feedstock	Objective	Parameter Changes	MMT CO_2e	USD/tCO_2e
Baseline	*MSW*	*Emis.; Cost*	*N/A*	*3.495; 2.469*	*$508.21; $367.16*
Conversion - Low	MSW	Emis.; Cost	waste to compost = .275	2.652; 1.682	$659.64; $474.56
Conversion - High	MSW	Emis.; Cost	waste to compost = .825	4.338; 3.074	$441.32; $302.22
Reduced Feedstock	MSW	Emis.; Cost	disposal fraction = 0.45	3.495; 1.485	$508.21; $326.96
Processing - High	MSW	Emis.	processing emis = 84.5	3.495	$520.70
Baseline +	*MSW + AR*	*Emis.*; *Cost*	*N/A*	22.097; 16.445	$726.24; $666.76
OF Cost - Low (+)	MSW; MSW + AR	Cost	Construction cost =9.5	2.463; 16.337	$810.73; $737.97
Land-use + Flow	MSW + AR	Emis.; Cost	Feedstock accepted: OF = AR; Comm. = Any	18.318; 15.244	$843.82; $826.41
EJ + Flow	MSW + AR	Emis.; Cost	Feedstock accepted: OF = AR; Comm. = Any	21.404; 16.037	$787.97; $717.19
Baseline + Flow	MSW + AR	Emis.; Cost	Feedstock accepted: OF = AR; Comm. = Any	22.314; 16.418	$675.99; $603.99
EJ & AQ + Flow	MSW + AR	Emis.; Cost	Feedstock accepted: OF = AR; Comm. = Any	20.184; 15.323	$902.23; $810.06

SI References

(1) CalRecycle. *SB 1383 Infrastructure and Market Analysis Report*; DRRR-2019-1652; CalRecycle, 2019; p 126. https://www2.calrecycle.ca.gov/Publications/Details/1652.
(2) Scown, C.; Breunig, H.; Kavvada, O.; Huntington, T. Biositing Webtool, V1, 2019. https://doi.org/10.11578/DC.20191029.4.
(3) Silver, W. L.; Vergara, S. E.; Mayer, A. *Carbon Sequestration and Greenhouse Gas Mitigation Potential of Composting and Soil Amendments on California's Rangelands*; California's Fourth Climate Change Assessment CCC4A-CNRA-2018–002; California Natural Resources Agency, 2018; p 62.
(4) Adhikari, B. K.; Barrington, S.; Martinez, J.; King, S. Characterization of Food Waste and Bulking Agents for Composting. *Waste Manag.* **2008**, *28* (5), 795–804. https://doi.org/10.1016/j.wasman.2007.08.018.
(5) Levis, J. W.; Barlaz, M. A. What Is the Most Environmentally Beneficial Way to Treat Commercial Food Waste? *Environ. Sci. Technol.* **2011**, *45* (17), 7438–7444. https://doi.org/10.1021/es103556m.
(6) CalRecycle. *SWIS Facility/Site Search*. Solid Waste Information System (SWIS). https://www2.calrecycle.ca.gov/SolidWaste/Site/Search (accessed 2024-03-26).
(7) CalRecycle. *Analysis of the Progress Toward the SB 1383 Organic Waste Reduction Goals*; DRRR-2020-1693; CalRecycle, 2020.
(8) Cotton, M.; Rynk, R.; Loder-Rossiter, L.; Carpenter, A. Site Planning, Development, and Environmental Protection. In *The Composting Handbook*; Elsevier, 2022; pp 409–500. https://doi.org/10.1016/B978-0-323-85602-7.00018-2.
(9) CDOC. *Farmland Mapping & Monitoring Program*. California Department of Conservation. https://www.conservation.ca.gov/dlrp/fmmp (accessed 2024-03-26).
(10) CDWR. *Statewide Crop Mapping - California Natural Resources Agency Open Data*. California Department of Water Resources. https://data.cnra.ca.gov/dataset/statewide-crop-mapping (accessed 2024-03-26).
(11) Don, A.; Seidel, F.; Leifeld, J.; Kätterer, T.; Martin, M.; Pellerin, S.; Emde, D.; Seitz, D.; Chenu, C. Carbon Sequestration in Soils and Climate Change Mitigation—Definitions and Pitfalls. *Glob. Change Biol.* **2024**, *30* (1), e16983. https://doi.org/10.1111/gcb.16983.
(12) Gravuer, K. *Compost Application Rates for California Croplands and Rangelands for a CDFA Healthy Soils Incentives Program*; California Department of Food and Agriculture, 2016. https://www.cdfa.ca.gov/oefi/efasap/docs/CompostApplicationRate_WhitePaper.pdf.
(13) CARB. *Quantification of Greenhouse Gas Emissions for Compost Application in California Croplands*; California Air Resources Board, Research Division, 2017. https://ww2.arb.ca.gov/sites/default/files/classic//cc/capandtrade/auctionproceeds/dndc_calculations.pdf (accessed 2020-12-17).
(14) CalEPA. *SB 535 Disadvantaged Communities*. OEHHA. https://oehha.ca.gov/calenviroscreen/sb535 (accessed 2024-03-26).
(15) Resource Recycling Systems. *District of Columbia Compost Feasibility Study*; District of Columbia Department of Public Works, 2017.
(16) EPA. Waste Transfer Stations: A Manual for Decision-Making. **2002**.
(17) Hall, A. L.; Potts, M. D.; Silver, W. L. Near-Term Potential of Organic Waste Management Infrastructure for Soil Carbon Sequestration in Rangelands. *Environ. Res. Infrastruct. Sustain.* **2022**, *2* (4), 045007. https://doi.org/10.1088/2634-4505/ac970f.

(18) Platt, B. *State of Composting in the U.S.: What, Why, Where & How*. Institute for Local Self-Reliance. https://ilsr.org/state-of-composting/ (accessed 2024-03-25).
(19) CARB. *Mobile Source Emission Inventory - EMFAC2017 Web Database*. California Air Resources Board. https://arb.ca.gov/emfac/2017/ (accessed 2020-10-16).
(20) Vergara, S. E.; Silver, W. L. Greenhouse Gas Emissions from Windrow Composting of Organic Wastes: Patterns and Emissions Factors. *Environ. Res. Lett.* **2019**, *14* (12), 124027. https://doi.org/10.1088/1748-9326/ab5262.
(21) Wernet, G.; Bauer, C.; Steubing, B.; Reinhard, J.; Moreno-Ruiz, E.; Weidema, B. The Ecoinvent Database Version 3 (Part I): Overview and Methodology. *Int. J. Life Cycle Assess.* **2016**, *21* (9), 1218–1230. https://doi.org/10.1007/s11367-016-1087-8.
(22) US EPA, O. *Waste Reduction Model (WARM)*. US EPA. https://www.epa.gov/warm/basic-information-about-waste-reduction-model-warm (accessed 2020-10-16).
(23) Breitenbeck, G. A.; Schellinger, D. Calculating the Reduction in Material Mass And Volume during Composting. *Compost Sci. Util.* **2004**, *12* (4), 365–371. https://doi.org/10.1080/1065657X.2004.10702206.
